# Circulating immune cells and risk of osteosarcoma: a Mendelian randomization analysis

**DOI:** 10.3389/fimmu.2024.1381212

**Published:** 2024-07-16

**Authors:** Lan Li, Yeqi Sun, Jia Luo, Mengjiao Liu

**Affiliations:** ^1^ Department of Pathology, The Second Xiangya Hospital, Central South University, Changsha, China; ^2^ Hunan Clinical Medical Research Center for Cancer Pathogenic Genes Testing and Diagnosis, Changsha, China; ^3^ Hunan Provincial Key Laboratory of the Research and Development of Novel Pharmaceutical Preparations, Changsha Medical University, Changsha, China; ^4^ Clinical Nursing Teaching and Research Section, The Second Xiangya Hospital, Central South University, Changsha, China

**Keywords:** osteosarcoma, immune cell, Mendelian randomization analysis, risk factor, immunotherapy

## Abstract

**Objectives:**

Osteosarcoma (OS) is the primary bone tumor originating from transformed mesenchymal cells. It is unclear whether associations between specific circulating immune cells and OS are causal or due to bias. To clarify whether predicted genetically altered circulating immune cells are associated with OS development, we performed a two-sample Mendelian randomization (MR) analysis.

**Methods:**

The genetic variants strongly associated with immune cell traits as instrumental variables (IVs) were used to perform MR analyses. The effect of specific immune cells on OS risk was measured using the summary statistics from the genome-wide association studies (GWAS).

**Results:**

Our findings indicate that CD80 on CD62L+ myeloid dendritic cell and CD28−CD4−CD8− T-cell absolute count are positively associated with OS (CD80 on CD62L+ myeloid dendritic cell, OR: 3.41 [95% CI: 1.40 to 8.31], *p* = 0.007; CD28−CD4−CD8− T-cell absolute count, OR: 4.49 [95% CI: 1.29 to 15.62], *p* = 0.018). It is also found that CD20 has a negative effect on CD24+CD27+ B cell on OS (OR: 0.32 [95% CI: 0.14 to 0.72], *p* = 0.006) and a similar impact on IgD+ CD38− B cell on OS (OR: 0.19 [95% CI: 0.05 to 0.68], *p* = 0.011).

**Conclusions:**

These findings illustrate that the genetic predisposition to specific immune cells can exert a causal effect on OS risk, which confirms the crucial role played by immunity in OS development. Particularly, the causal association between immune cells and OS underscores the evidence for exploring the new treatment strategy for OS in the future.

## Introduction

Osteosarcoma (OS) is a malignant primary bone tumor originating from transformed mesenchymal cells that usually occurs in children, adolescents, and young adults (1, 2). Moreover, the 5-year survival rate for recurrent or metastatic patients of osteosarcoma is less than 25% (1, 2). In order to uncover the etiology of osteosarcoma, subsets of studies show that DNA repair, growth regulation, antigen processing and presentation, and telomere maintenance pathways were associated with an increased risk of osteosarcoma (2). Innate immunocytes, one of the vital effector cell subpopulations, could directly recognize and kill, or self-activate, to trigger a strong adaptive immune response in cancer. (3). However, the prognosis of osteosarcoma patients is still poor due to drug resistance, metastasis, or recurrence. Recently, observational evidence has indicated that the immune cells play an important role in the poor outcome of osteosarcoma patients, which includes drug resistance and metastasis (4–6). Therefore, it is essential to explore further the highly specialized immune cells related to osteosarcoma genes to identify potential therapeutic biomarkers and targets to improve the prognosis of osteosarcoma.

Previous studies have indicated that the immune cells are connected with the malignant activity of osteosarcoma. The recruitment and differentiation of immune infiltrating cells, including T lymphocytes, macrophages, and other subpopulations, such as B lymphocytes and mast cells, was conducive to tumor growth, drug resistance, and metastasis. For example, lower infiltration of CD8-positive T cells was associated with the proliferation of osteosarcoma cells (7). Enriched M2 macrophages in primary osteosarcoma tissues may closely relate to drug resistance and the activation of cancer stem cells (8). While the regulation of immune cells for inhibiting cancer progression has been much discussed in cancer therapy, few studies have illustrated the causal association between circulating immune cells and osteosarcoma onset. Furthermore, large-scale randomized clinical trials are expensive, and observational analysis might induce potential confounders and unmeasured reverse causality. If the associations between immune cells and osteosarcoma are clarified, the newer immune-based treatments, including immune checkpoint inhibitors, vaccination, and adoptive cell therapy, may widely improve treatment effects for cancer therapy in the future.

Since genetic variants are randomly assigned at conception prior to disease onset, Mendelian randomization (MR) as an efficient tool can exploit these variants as unbiased proxies to approximate the estimation of an exposure effect on the outcome of interest while minimizing the effects of confounding and reverse causation (9). To date, longer leukocyte telomere lengths (LTLs) are causally associated with osteosarcoma via the MR approach (2). However, few MR studies have addressed the causal relationship between circulating immune cell traits and osteosarcoma. The purpose of this study was to determine whether there is a causal connection between circulating immune cells and osteosarcoma incidence. Understanding the risk factors associated with osteosarcoma progression will aid in the development of novel therapy strategies to reduce cancer risk.

## Materials and methods

### Study design

**Figure 1:**
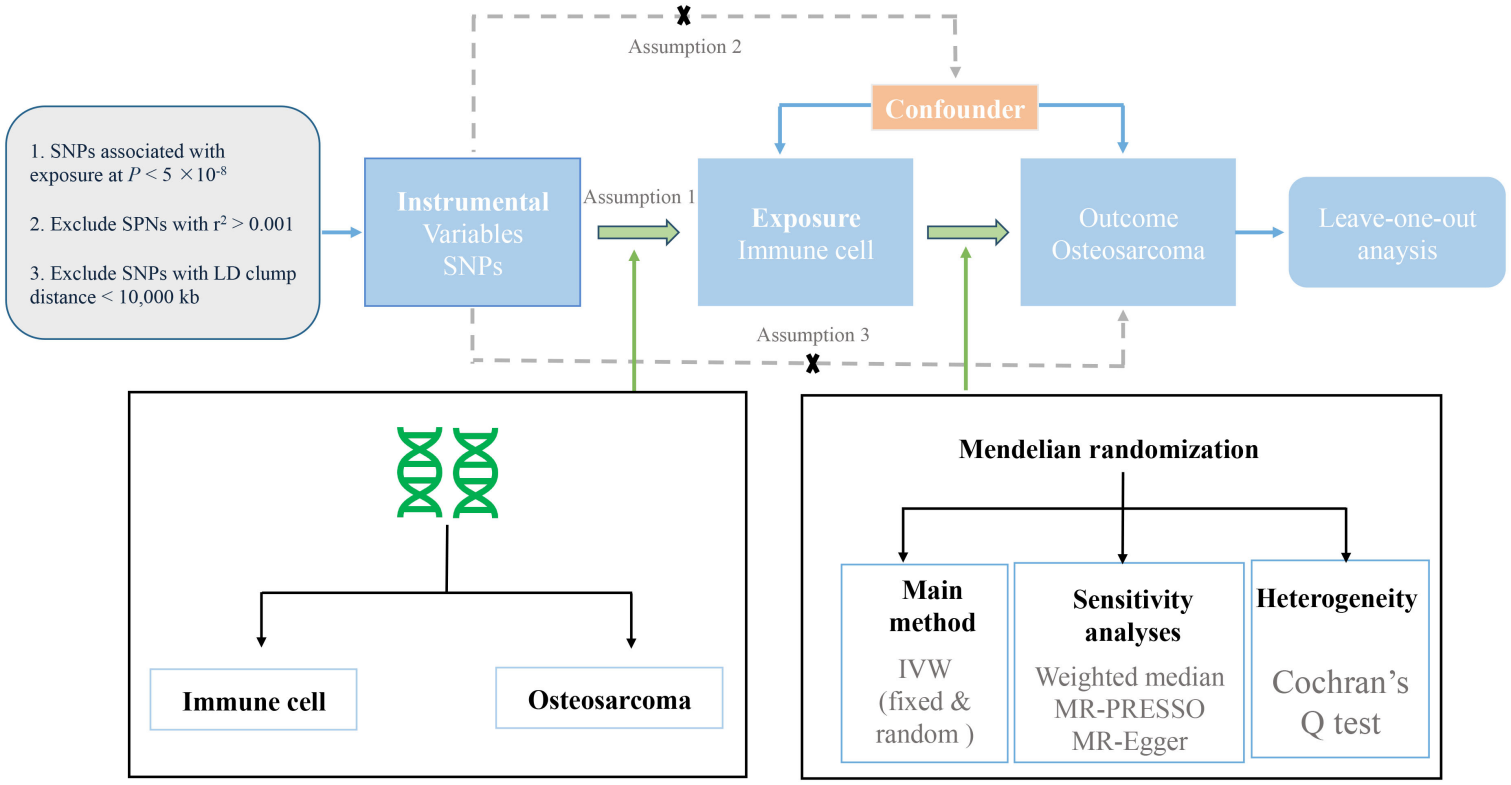
The overview of instrumental SNP selection and Mendelian randomization (MR) analysis.

We evaluated the causal relationship between 731 immune cell characteristics (seven groups) and osteosarcoma based on MR analysis. MR is based on three key assumptions (10): (1) Genetic variation was strongly associated with exposure; (2) Instrumental variable (IV) has nothing to do with confounding factors; and (3) Genetic variation does not directly affect the results but may indirectly affect the results through exposure (2). In this study, we first performed MR analysis to analyze the effect of immune cells on the risk of osteosarcoma. A series of sensitivity analyses were further used to evaluate the robustness of the results. The design process of this study is shown in **Figure 1**.

### Data sources

For immune cell traits, we sourced summary statistics from a meta-analysis of genome-wide association studies (GWAS), covering 3,757 Sardinian cohort participants (11). A total of 731 immunophenotypes were identified, including absolute cell (AC) count (*n* = 118), median fluorescence intensity (MFI) (*n* = 389), morphological parameter (MP) (*n* = 32), and relative cell (RC) count (*n* = 192) (10). For osteosarcoma, association data originated from a FinnGen consortium GWAS (R10) involving 64 cases and 314,193 controls, incorporating genetic and health data from around 500,000 participants (12). The diagnosis criteria of osteosarcoma were consistent with the International Classification of Diseases Code 10 (ICD-10), and Illumina and Affymetrix arrays were used for genotyping. A total of 20,169,975 SNPs were analyzed (11). This GWAS was adjusted for gender, age, and the first 10 principal components of ancestry (11).

The instrumental variable (IV) in the MR analysis of this study was single-nucleotide polymorphism (SNP), which was significantly associated with exposure and outcome. In order to increase the statistical power of genetic variation, we conducted a series of quality control steps to select qualified SNPs. The details of the data source we used are listed in **Supplementary Table S1**.

### Selection of instrumental variables

For immune cell IVs, we initially chose them per cell using a stringent *p* < 5 × 10^−8^. Next, the independent IVs with the lowest *p*-value for each trait (*r* ^2^ < 0.001 and distance = 10,000 kb) were retained to reduce the influence of correlations among SNPs, leaving a total of 6,879 SNPs (**Supplementary Table S2**). Since 1,240 SNPs were not available in the outcome dataset of osteosarcoma in this current study, 5,639 SNPs were associated with 477 immune cell traits in the subsequent MR analysis. Utilizing publicly available GWAS summary data and IRB-approved studies with participant consent, our study did not necessitate further ethical clearance.

### Statistical analysis

First, we calculated the F-statistics to assess the strength of genetic instruments and to examine whether the effect estimates of the causal associations were likely to be affected by weak instrument bias. F-statistics were calculated using the following formula: F-statistics = (Beta/Se)^2^, and its mean was regarded as the overall statistics (13). F-statistics > 10 indicated strong statistical power (14).

In this study, we used the inverse-variance weighted (IVW) method as the primary analysis. After extracting the association estimates linking the instruments and outcomes and harmonizing the directional orientation of these estimates as the effect alleles, we applied the Wald estimator in our computation of MR estimates for each instrument, which allowed us to derive estimates of the causal effect. We performed Cochran’s *Q* test to estimate the heterogeneity among SNPs (15). The random-effects model was employed if significant heterogeneity was presented (*p* < 0.05); otherwise, the fixed-effects model was used (14). To calculate the Wald estimates, effect estimates of each SNP on immune cell traits and the risk of osteosarcoma were obtained by the IVW method (16). When invalid IVs are present, they are susceptible to the influence of the potential pleiotropic effects (15). Therefore, we conducted a series of sensitivity analyses to assess the robustness of the results. First, we applied the weighted median method to provide a reliable estimate, which assumes that at least 50% of weights were derived from the valid IVs (17). Second, the maximum-likelihood method was used, which may display a more credible association result when measurement error exists in the SNP-exposure effects (18). Third, we used MR pleiotropy residual sum and outlier test (MR-PRESSO) to test and correct for potential outliers (19). At last, we used MR-Egger regression to detect directional pleiotropy by evaluating whether the intercept was statistically different from zero (20). Weighted mode was also used as a supplementary analysis method, which is sensitive to the difficult bandwidth selection for mode estimation (21). Finally, we also conducted a leave-one-out analysis to test the stability of the causality after excluding SNPs one by one.

Unless otherwise specified, all statistical analyses were conducted using the R software (version 4.1.1). The “Mendelian Randomization” and “TwoSample MR” R packages were utilized in the study. Odds ratios (ORs) and 95% confidence intervals (Cis) were computed to assess the association between immune cell traits and osteosarcoma. Statistical significance was assigned to two-sided *p*-values less than 0.05.

## Results

**Figure 2:**
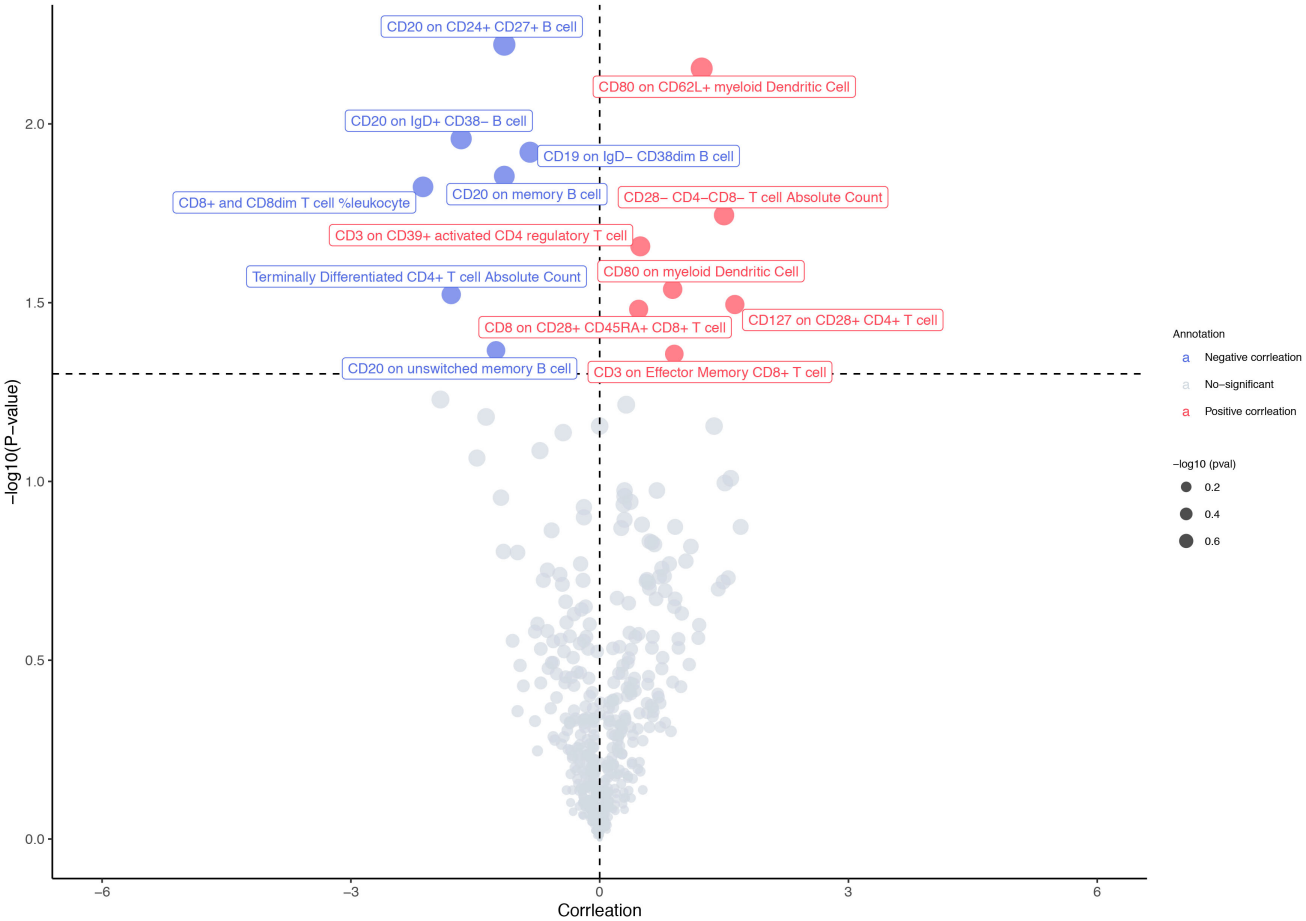
MR results in primary analysis. The IVW method identified 14 immune cell traits with a significant association to osteosarcoma. Among these 14 immune cell traits, seven were positively associated with the osteosarcoma risk, while the other seven were inversely related to the risk of osteosarcoma.

**Figure 3:**
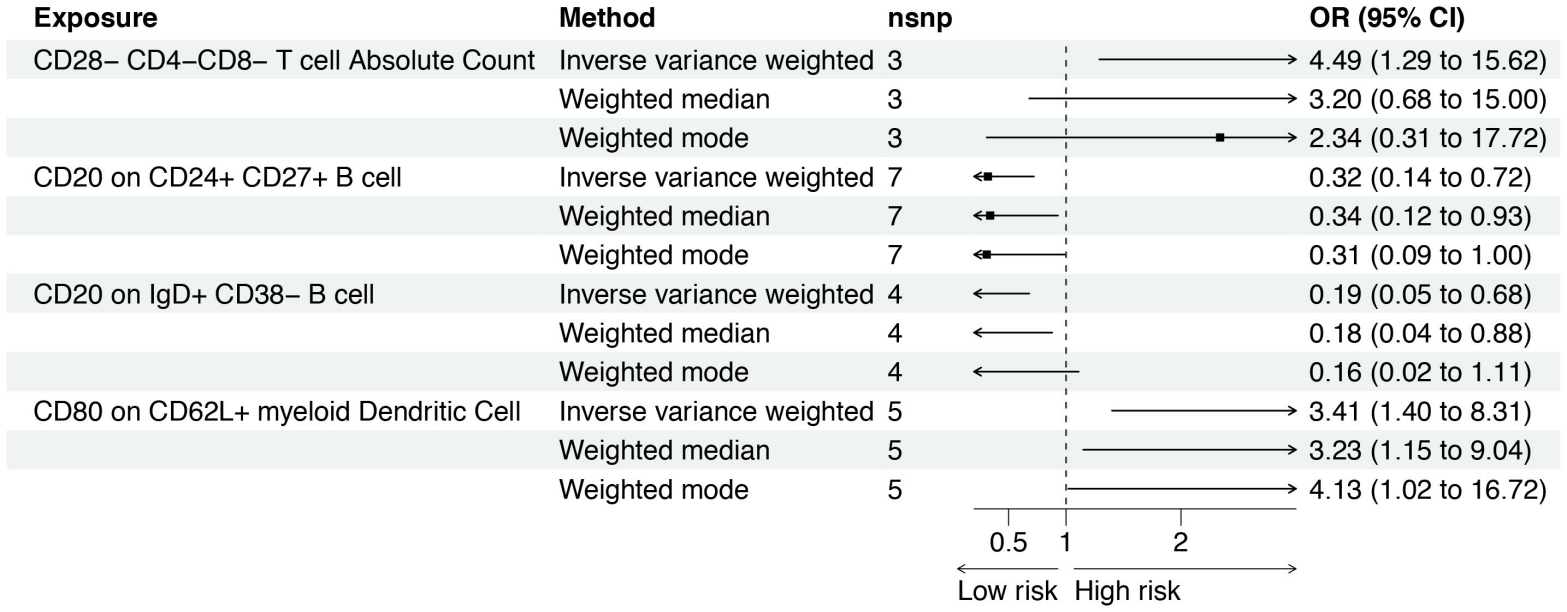
Forest plot for the causal effect of circulating immune cells on the risk of osteosarcoma derived from inverse variance weighted (IVW), weighted median, and IVW radial. OR, odds ratio; CI, confidence interval; OS, osteosarcoma.

The F-statistics of IVs ranged from 29.84 to 3161.45, suggesting that the association between immune cell traits and osteosarcoma would not be influenced by weak instruments (**Supplementary Table S2**). Out of the 477 immune cell traits analyzed, we identified 14 that showed significant associations with osteosarcoma using the IVW method. Among these 14 traits, seven were positively associated with the risk of osteosarcoma, while the remaining seven exhibited an inverse relationship with this risk (**Figure 2**). Specifically, CD80 expression on CD62L+ myeloid dendritic cells (OR: 3.41 [95% CI: 1.40 to 8.31], *p* = 0.007) and the absolute count of CD28−CD4−CD8− T cells (OR: 4.49 [95% CI: 1.29 to 15.62], *p* = 0.018) were found be positively associated with osteosarcoma in the IVW analysis. The causality between CD80 on CD62L+ myeloid dendritic cell and osteosarcoma risk remained consistent across other methods, such as weighted median and weighted mode (**Figure 3**). Moreover, there was no heterogeneity among the selected SNPs, as indicated by all *p*-values for Cochran’s *Q* test > 0.05 (**Supplementary Table S3**). The intercept of MR-Egger did not reveal any pleiotropy of variants in the estimated associations (all *p* > 0.05, **Supplementary Table S4**). Furthermore, the results of the leave-one-out analysis indicated that the associations were not influenced by any single IVs (**Supplementary Figure S1A**).

There was a negative effect of CD20 on CD24+CD27+ B cells on osteosarcoma based on the IVW (OR: 0.32 [95% CI: 0.14 to 0.72], *p* = 0.006). As shown in **Figure 3**, this causality was observed in weighted median methods (OR: 0.34 [95% CI: 0.12 to 0.93], *p* = 0.035). The MR-Egger results suggested no influence of IVs pleiotropy in the MR analysis (*p* = 0.267). However, the results of the weighted mode did not observe a similar association (*p* = 0.099). CD20 on IgD+ CD38− B cells also have a similar impact on osteosarcoma (OR: 0.19 [95% CI: 0.05 to 0.68], *p* = 0.011), and the weighted median method supported it (**Figure 3**). However, the leave-one-out analysis suggested that the association slightly fluctuated after excluding rs30003 (**Supplementary Figure S1B**). More details about the association between immune cell traits and osteosarcoma are presented in **Supplementary Table S5**.

## Discussion

The MR analysis has been commonly applied to investigate the potential causal link between risk factors and diseases. With the aid of MR analyses, we were able to estimate the potential causal relationship between the circulation of immune cells and the risk of osteosarcoma from a genetic perspective. Our findings found that CD80 on CD62L+ myeloid dendritic cells and CD28−CD4−CD8− T-cell absolute count are positively associated with OS, and CD20 on CD24+CD27+ B cells and CD20 on IgD+ CD38 B cells have a negative effect on osteosarcoma. It suggested that circulating immune cell subtypes could play both a promotive and protective role in the risk of osteosarcoma.

Consistent with our study, previous experimental and retrospective studies have indicated a strong association between the percentage and activity level of certain peripheral blood immune cell subtypes and the risk of osteosarcoma (22–25). A recent systematic review investigating the myeloid-derived immune suppressor cell (MDSCs) subtypes and the prognosis of non-small cell lung cancer (NSCLC) patients also showed evidence that patients with high monocytic MDSC levels in peripheral blood experienced a worse prognosis (26). Indeed, similar MR analysis has reported a protective causal effect of elevated levels of circulating lymphocyte counts on colorectal cancer risk, though in other cancers and not involving specific immune cell subsets (27). In another systematic review, it was found that the high CD8+ tumor-infiltrating lymphocytes (TILs) were significantly associated with better prognosis in pan-cancer patients treated with immune checkpoint inhibitors. However, such tendency could not be observed with circulating CD8+ T cells from peripheral blood (28). A reason for this discrepancy could be a potential pleiotropic effect that the specific immune cells have depending on tumor type and stage. Undoubtedly, clarifying the functions of the circulating immune cell subtype in the pathogenesis and deterioration of osteosarcoma is important to provide novel ideas for improving clinical treatment. Utilizing publicly available genetic data, we performed a two-sample MR to investigate the effects of selected 477 circulating immune cell traits on the risk of osteosarcoma. The IVW method identified 14 immune cell traits with a significant association with osteosarcoma, which were then preliminarily screened out. Among these, four immune cell traits were finally identified as causal variables that have a dominant influence on osteosarcoma risk after a series of supplementary and sensitive analyses (weighted median, weighted mode, MR-Egger, MR-PRESSO, etc.). To our knowledge, this is the first prospective MR study that examined the association between circulating immune cells and osteosarcoma risk.

This study illustrated that increased CD80+CD62L+ myeloid dendritic cells appeared to be linked with osteosarcoma risk. CD80 and CD86, two identified members of the B7 family proteins expressed on dendritic cells, B cells, and antigen-presenting cells, could bind to costimulatory molecules CD28 and CTLA4 and act as their ligands to provide second signals for efficient activation or inhibition of T cells (29). According to the literature, CTLA4 with a higher binding affinity to CD80 delivers inhibitory signals for activation of T lymphocytes and immune responses against malignant tumors (27). Findings from an observational study have shown that increased CTLA4 T cells were found in aggressive pediatric osteosarcoma patients (30). Using single-cell sequencing technology, another observational study revealed that exhausted T cells exhibited substantial infiltration and a progressive increase in expression of CTLA4 in osteosarcoma samples. (31). An experimental study found that infection upregulates the inflammatory immune response mediated by canine macrophages, counteracting osteosarcoma-induced immune suppression through increasing CD80 expression (32). Inconsistent results here may be due to studies carried out on different species and changes in the leading interaction between CD80 and CD28 or CTLA4 in certain circumstances. In sum, enhanced CD80-CTLA4 crosstalk-mediated immunosuppressive phenotypes of T cells may play vital roles in regulating osteosarcoma biological process. As for CD62L, although there’s limited literature support, its reported function, known as accelerating cell migration and intercellular contact, might provide insight into our findings of this tumor-promoting effect on osteosarcoma (33).

Our data also showed an increase in absolute cell counts of CD28−CD4−CD8− T cells correlating with the onset of osteosarcoma. As mentioned, CD28 strongly binding to CD86 delivers stimulation signals to immune response and induces survival of T lymphocytes (20). In support of this result, a biomarker experiment study showed that the CD86/CD28 stimulatory pathway associated with activated memory CD4 T cells was dominant in osteosarcoma patients with a good prognosis (34). Thus, decreased expression of CD28 could lead to disability of the activation function of T cells and help to shape an immunosuppressive phenotype for osteosarcoma progression (1). Moreover, CD4−CD8-double-negative T cells (DN T) are considered important members of innate and adaptive immune systems, although they represent a small fraction of circulating T lymphocytes (35). Many tumor-related studies have tried to illustrate the impact of DN T cells on various types of tumors and found that DN T possessed protumor or antitumor effects depending on the tumor type. It has been shown that DN T not only exists in the peripheral blood but also infiltrates solid tumors such as non-small-cell lung cancer, liver cancer, glioma, and pancreatic tumors. One observational study suggested that the change in peripheral DN T during treatment with checkpoint inhibitors in metastatic melanoma patients may be adept at sensing the immune response to melanoma. To date, no report has been found in the literature devoting the possible relationship between DN T and osteosarcoma. Therefore, this result may provide a new avenue for investigating the immunological mechanisms of DNT in osteosarcoma and could ultimately provide novel knowledge for understanding this disease.

The research also identified a negative association between CD20+CD24+CD27+ B cells and CD20+IgD+CD38− B cells with osteosarcoma risk. The literature contains few reports of these two immune B-cell subtypes. Recently, an emerging focus on the function of peripheral blood B cells and tumor-infiltrating B lymphocytes has uncovered their crucial, synergistic role in modulating tumor immunoenvironment (36, 37). The recent discovery found of B10 cells exemplifies this concept. These cells likely contribute to an antitumor immunity effect by producing IL-10 and inducing the generation of immunosuppressive T cells. In some observational studies, both greater infiltration of the tumor tissues by B10 cells and a high proportion of circulating B10 cells could result in a worse prognosis or tumor progression in gastric cancer, colorectal cancer, and cervical cancer (38–40). More than that, it was proposed that immune B cells exhibited a double role in promoting and inhibiting cancer progression depending on their phenotype (41). It is evident that deeper thinking and concern about the immune subtype of B cells in tumor etiology are necessary.

There are several limitations to this study. First, all analyses were performed using only European participants, making it difficult to assess whether these results can be extrapolated to other populations. Second, we can only access GWAS data for osteosarcoma compared to healthy individuals, and we cannot find GWAS summary statistics for osteosarcoma classified according to histopathological subtypes. Further MR analysis of the causal relationship between circulating immune cells and osteosarcoma subtypes will provide more evidence-based guidance for clinical practice. Third, it appeared that the results concerning two protective B-cell subtypes were not sufficiently robust compared to those of the promotive T-cell subtypes. Therefore, analyses of these protective B-cell subtypes should be repeated as larger GWAS become available. Fourth, it is known that the occurrence and development of osteosarcoma may be affected by various factors. Beyond focusing solely on outcome variables, it would be essential in future studies to explore pivotal variables associated with disease progression in OS patients, such as the tumor stage, metastasis status, and blood markers like alkaline phosphatase (ALP), lactate dehydrogenase (LDH), etc. This approach can help uncover causal inferences between circulating immune cells and the biology of osteosarcoma progression.

## Conclusions

The results generated here provide evidence for the dual role of circulation immune cells and indirectly corroborate the complexity of immune regulation in osteosarcoma. Additional research is needed to explore the underlying biological mechanisms of these certain immune cells and unravel the molecular basis of their interaction with tumor cells on osteosarcoma development. A better understanding of their function may facilitate further digging into rational biomarkers and designing new strategies that efficiently target these cells by immunotherapy.

## Data availability statement

The datasets presented in this study can be found in online repositories. The names of the repository/repositories and accession number(s) can be found in the article/**Supplementary Material**.

## Ethics statement

Ethical approval was not required for the study involving humans in accordance with the local legislation and institutional requirements. Written informed consent to participate in this study was not required from the participants or the participants’ legal guardians/next of kin in accordance with the national legislation and the institutional requirements.

## Author contributions

LL: Writing – original draft, Writing – review & editing, Formal analysis. YS: Formal analysis, Writing – original draft, Writing – review & editing. JL: Formal analysis, Writing – review & editing. ML: Formal analysis, Writing – review & editing.

## Supplementary material

Supplementary Figure 1
**(A)** MR leave-one-out sensitivity analysis for CD80 on CD62L+ myeloid dendritic cell (id: ebi-a-GCST90002036) on outcome. **(B)** MR effect size for CD20 on IgD+ CD38-B cell (id: ebi-a-GCST90001748) on outcome.
