# Supplementary figures and images for: Circulating immune cells and risk of osteosarcoma: a Mendelian randomization analysis
Source: Front Immunol. 2024 Jul 16;15:1381212. doi: 10.3389/fimmu.2024.1381212 (PMC11286390; doi:10.3389/fimmu.2024.1381212)

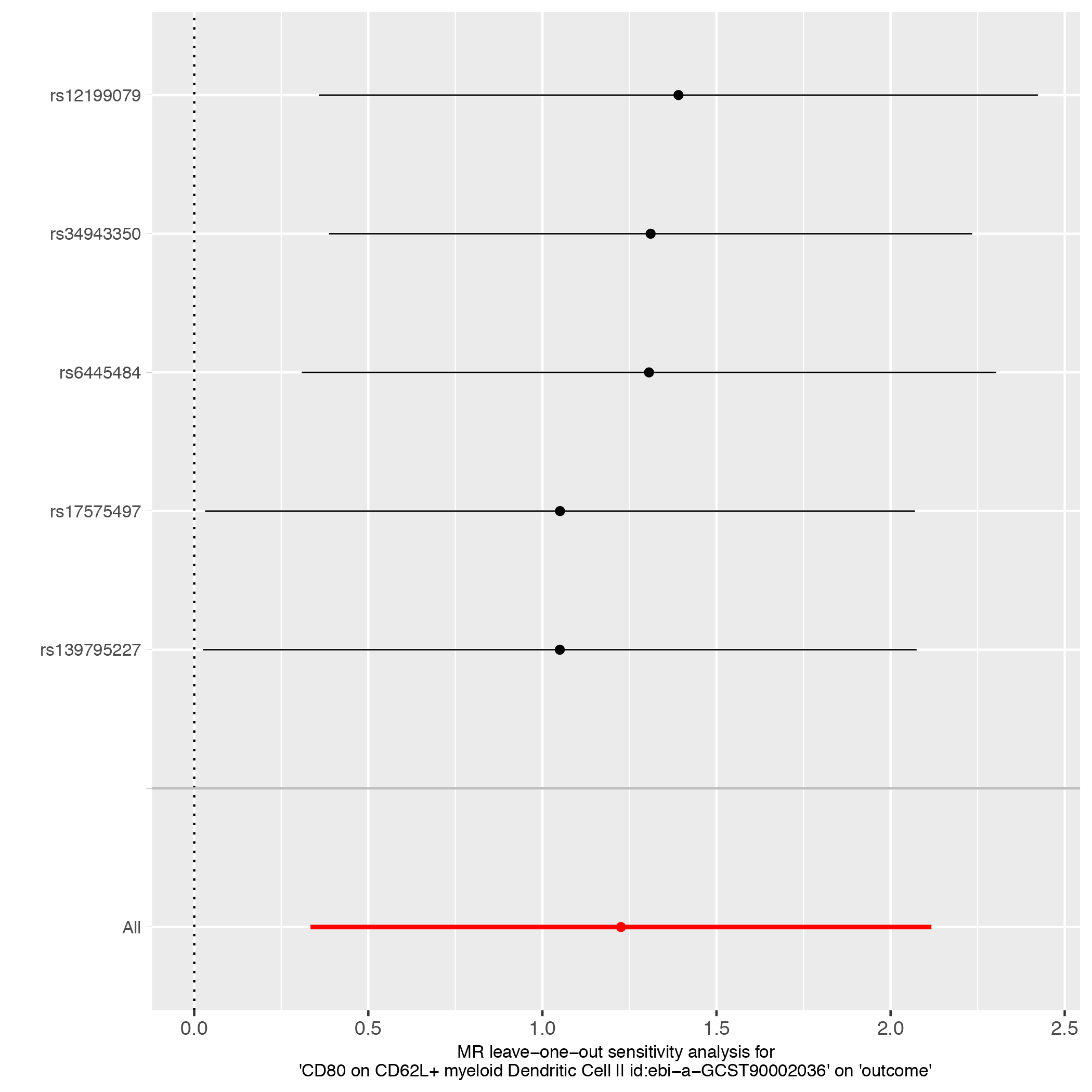

Supplement: Supplementary Figure 1 — (A) MR leave-one-out sensitivity analysis for CD80 on CD62L+ myeloid dendritic cell (id: ebi-a-GCST90002036) on outcome. (B) MR effect size for CD20 on IgD+ CD38-B cell (id: ebi-a-GCST90001748) on outcome. [file Image_1.tif]

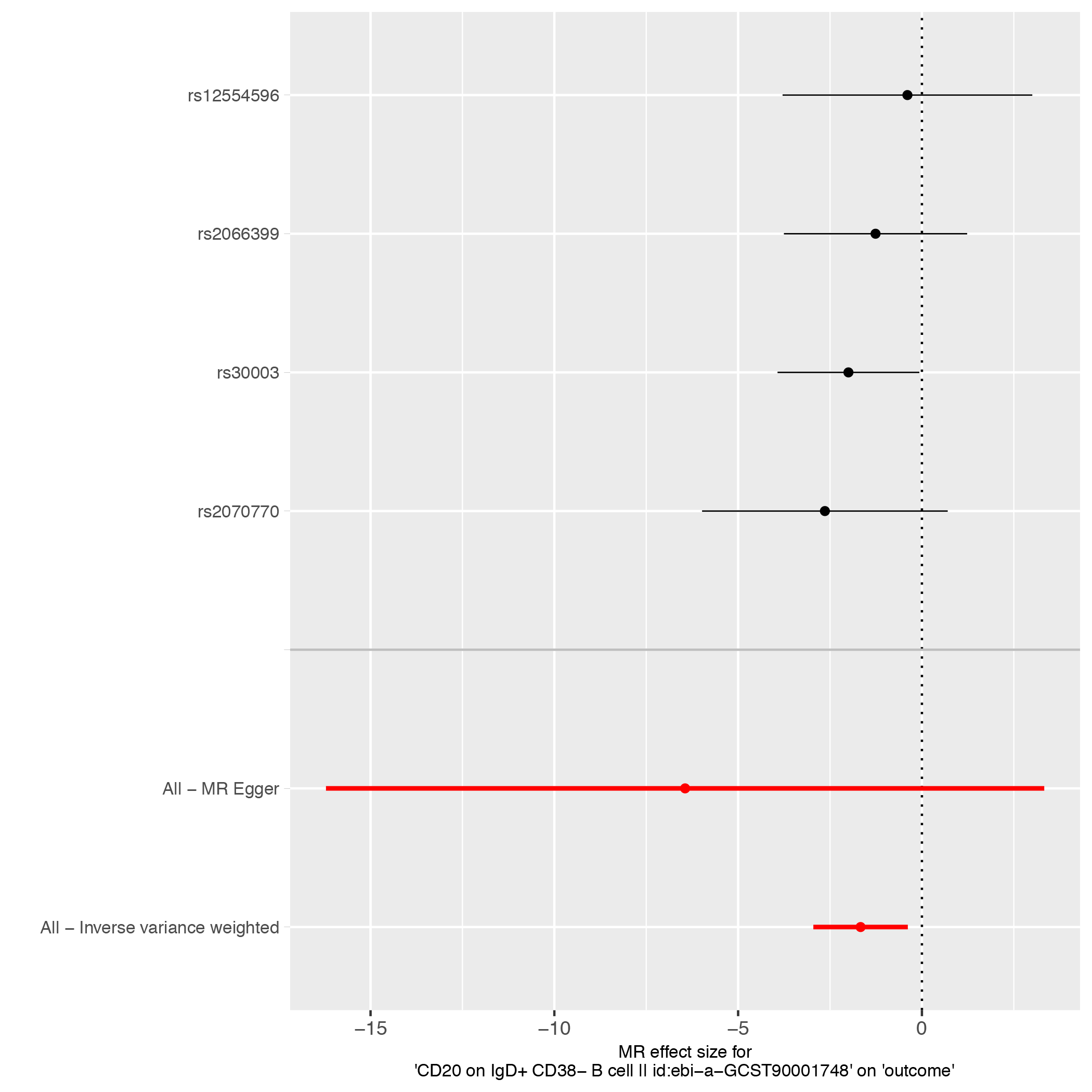

Supplement: Supplementary file 2 [file Image_2.tif]
